# Implementation Status and Challenges Associated With Implementation of the Targeted Medication Safety Best Practices in a Tertiary Hospital

**DOI:** 10.7759/cureus.45552

**Published:** 2023-09-19

**Authors:** Hamzah N Alothmany, Douha F Bannan

**Affiliations:** 1 School of Pharmacy, King Abdulaziz University, Jeddah, SAU; 2 Department of Pharmacy Practice, King Abdulaziz University Faculty of Pharmacy, Jeddah, SAU

**Keywords:** medication error, challenges, implementation rate, hospitals, best practices, medication safety

## Abstract

Background

The Institute for Safe Medication Practices (ISMP) is a well-known non-profit organization dedicated to preventing medication errors. Every two years they publish best practices that can reduce the occurrence of medication errors. This study aims to evaluate the implementation status of these best practices and to understand barriers associated with non-implementation at a tertiary hospital in Saudi Arabia.

Methodology

This was a two-phase qualitative study. First, a survey consisting of the ISMP best practices was sent to employees (mainly heads of departments) to fill out the implementation rate for each best practice. Then an interview or a focus group was conducted to further validate their answers and understand why some best practices were not implemented.

Results

Our study found that the highest implemented best practices were having strategies to improve safety with high-alert medications (best practice #19, 85.7%), having antidotes and reversal agents readily available (best practice #9, 75%), independent verification of sterile preparation (best practice #11, 75%), and limiting the number of removable medications from the automated dispensing unit by override (best practice #16, 75%). The least implemented best practices were ensuring that oral liquid medications are dispensed in a syringe (best practice #4, 12.5%), maximizing use of barcode verification (best practice #18, 12.5%), purchasing oral liquid dosing devices that display metric scale (best practice #5, 25%), eliminating glacial acetic acid from all areas of the hospital (best practice #6, 28.6%), and eliminating all 1,000 mL of sterile water from all areas outside of the pharmacy (best practice #10, 28.6%). Challenges associated with implementation were related to knowledge, motivation, and opportunity in the environment, with the latter being the highest barrier associated with non-implementation.

Conclusions

Healthcare providers need to have knowledge about the best practices and the rationale behind them, the motivation to perform them, and the necessary resources to implement the best practices in their hospital.

## Introduction

Medication error (ME) is one of the major issues that threaten the safety of patients in the hospital [1]. It is defined as “Any preventable event that may cause or lead to inappropriate medication use or patient harm while the medication is in the control of the health care professional, patient, or consumer. Such events may be related to professional practice, health care products, procedures, and systems, including prescribing, order communication, product labeling, packaging, and nomenclature, compounding, dispensing, distribution, administration, education, monitoring, and use” [1,2].

ME is the eighth leading cause of amendable and preventable deaths in the United States causing about 225,000 deaths per year [1,3]. Studies in Saudi Arabia (SA) have shown that the incidence of ME in hospitals is approximately 44.4% [4]. ME is considered a major burden on hospitals and the healthcare system in terms of wasted resources and increasing length of stay, costing the healthcare system approximately 42 billion dollars a year [1,4-6].

Worldwide, ME is one of the leading causes of avoidable harm [7]. In the last couple of years, a growing emphasis has been placed on patient and medication safety. Many guidelines, recommendations, and best practices have been published to help healthcare providers and hospitals deliver safe and effective care without harming patients such as the American Society of Health-System Pharmacists, Ministry of Health, and Food and Drug Administration [8-10].

One of the most notable organizations that work on medication safety is the Institute for Safe Medication Practices (ISMP). The ISMP is a well-known and respected non-profit organization devoted entirely to medication safety information. Every two years, the ISMP publishes the Targeted Medication Safety Best Practices for Hospitals. These best practices focus on avoidable errors that continue to occur and can be prevented by changes in practice. Therefore, the implementation of these best practices might reduce medication errors. When the list of best practices is updated every two years, some best practices stay on the list if implementation is still high. Others are archived when the implementation rate is high. Some best practices are added and labeled as new. In 2021, the ISMP conducted a survey to assess the implementation status of the 2020-2021 best practices in 156 large hospitals in the United States. The findings showed that the rate of implementation increased for most of the best practices (best practice #1, 2, 4, 5, 7, 9, 13, 14, 15, and 16), stayed the same for best practice #3, slightly decreased for best practices #10 and #1, and decreased by 13% for best practice #8. The overall percentages of implementation rate for each best practice varied and ranged from 30% to 95%. The ISMP concluded that hospitals should focus on the implementation of the best practices in the following two years. As for the newest best practices (#17-19), the ISMP found that there is still room for improvement regarding their implementation rate [11-13].

In SA, we conducted a service improvement project in 2020 in a tertiary hospital to measure the awareness of pharmacy staff about the ISMP best practices 2018-2019. Almost all participants interviewed except one (13/14, 93%) did not know what the ISMP best practices are. Because participants were not given pre-specified options for the implementation status, the answers varied among “yes,” “no,” “I don’t know,” “I guess,” and “it depends.” The project showed a great area for improvement. However, it was not published as a separate paper due to a lack of standardization in the data collection methods. To our knowledge, no study has previously been conducted in SA to measure the implementation status of the targeted medication safety best practices. This study aimed to assess the implementation status of the ISMP best practices in a tertiary hospital in SA and the challenges associated with non-implementation.

## Materials and methods

Study design

This was a two-phase, prospective, qualitative, single-center study using a survey followed by either an interview or a focus group with the pharmacy managers to measure the implementation status of the ISMP Targeted Medication Safety Best Practices and understand challenges associated with non-implementation.

Study setting

The study was conducted at the pharmacy department of a tertiary care hospital in SA. It is a large hospital in the western region of SA with a capacity of more than 1,000 beds [14]. The Pharmaceutical Services Department in the hospital provides pharmaceutical care to patients through three pharmacies, namely, in-patient pharmacy, out-patient pharmacy, and satellite pharmacies. The Pharmaceutical Services Department employs approximately more than 100 healthcare providers, eight of whom hold managerial positions. Healthcare providers included in this study were the eight managers such as the director of the department and supervisors in charge of each service in the pharmacy (e.g., in-patient, and out-patient services). Our target was only managers and not all staff of the pharmaceutical department because their positions granted them more knowledge of the implementation status for each best practice compared to other employees.

Survey

The survey we used was developed by the ISMP to help hospitals assess the level of their current implementation status and the challenges associated with the ISMP Targeted Medication Safety Best Practices for Hospitals (TMSBP). It is a detailed worksheet that asks about the implementation status of 19 best practices including the subsets for each best practice. The ISMP updates and publishes the list of best practices every two years. The ISMP keeps the number of each best practice the same in all published versions, even if the best practice is archived. In the 2022-2023 version (used in this paper), four best practices (best practices #4, 5, 6, 10) were archived compared to the previous version (2020-2021). One best practice (best practice #12) was incorporated into best practice #15 [15]. Refer to Appendix A for definitions of each best practice.

The survey provides four options related to the implementation status for each practice (fully implemented, in progress, not implemented, or not applicable) [13]. The worksheet did not have a definition for these options. Therefore, we used the operational definitions listed in Table 1 during the survey and the interview/focus group.

**Table 1:** Operational definitions of the implementation status options.

Implementation status	Operational definition
Fully implemented	The best practice has already been implemented
In progress	The process of implementation started, but the best practice is not fully implemented yet
Not implemented	The best practice has not been implemented
Not applicable	The best practice is not applicable to the hospital, or the best practice does not apply to a particular situation in the hospital

Data collection

Participants were reached by contacting them directly to fill out the ISMP survey beforehand. We explained to them the aims of the study, what the survey is assessing, and that we will reach them after they fill out the survey to meet with them and discuss challenges related to implementation. Upon agreement to participate, the survey was sent to them via email or text message (whichever way they preferred). Reminders were sent to participants who did not fill out the survey at first, and when all participants had filled out the survey, we contacted them again to conduct a focus group or an interview.

Interview/Focus group

After the participants filled out the survey, they were invited to meet with the research team to discuss the findings from the survey and elaborate more on challenges related to the implementation of best practices. The choice of a one-to-one interview or focus group was mainly based on the availability of the participants. If several participants were available at the same time, then a focus group was conducted, if not then a one-to-one interview was the other option.

At the time of the meeting, we reintroduced the aims of the study, went through the participant information sheet (Appendix B), and then asked participants to sign the consent form (Appendix C). All participants were assured that participation was voluntary and that they were free to decide whether they wanted to be part of the study or not. Researchers displayed the implementation status of each best practice based on the findings of the survey. Participants were then asked to elaborate more on the findings and challenges related to non-implementation. The interview schedule is available in Appendix D. All discussions were audio-recorded and transcribed verbatim. The transcripts were coded independently by the authors. In case of disagreement, the researchers re-read the manuscript, re-discussed the issue, and clarified it with the participants, if needed.

Data analysis

Descriptive statistics were performed to summarize data such as the implementation status. Thematic analysis was used to identify themes related to challenges. Ethical approval was granted by the Institutional Review Board at the institution (PH-104-41).

## Results

Baseline characteristics

All managers (n = 8) filled in the questionnaire and five of them attended either an interview or a focus group. The remaining participants (n = 3) were not able to attend any discussion due to their unavailability. All participants had over five years of experience in the institution and held managerial positions in the hospital. Refer to Table 2 for complete baseline characteristics.

**Table 2:** Characteristics of participants.

	Participant (n)	Percentage (%)
Age (30–60 years old)	8	100%
Male	3	37.5%
Female	5	62.5%
Over five years of experience	8	100%
One-to-one interview	2	25%
Focus group	1 (with 3 participants)	37.5%

Survey findings

The best practices with the highest implementation rate were best practice #19 (85.7%) and best practices #9, #11, and #16 (75% each). The best practices with the least implementation rate were best practices #4 and #18 (12.5% each), #5 (25%), #6 and #10 (28.6% each). For best practice #10, an equal number of participants believed the best practice was fully implemented versus not implemented (37.5% each). Refer to Appendix E for more detailed information.

Interview/Focus group findings

During the discussions, the participants explained the reasons for non-implementation. They also clarified that their understanding of the option “not applicable” in the survey was that the best practice cannot be implemented due to limitations in the hospital system or lack of resources. Therefore, participants reclassified all the “not applicable” as “not implemented” and elaborated more on the challenges. After analyzing all the challenges, they were classified into three themes, namely, challenges related to knowledge, motivation, and opportunity.

Knowledge

Lack of knowledge such as not knowing the importance of or rationale behind implementing the best practices was one of the challenges that affected implementation of best practices. For example, many participants did not know why it was needed to have a default weekly order for oral methotrexate (best practice #2).

“Why is it a problem if this happens?” Participant (P) 3, Focus group (FG) 1.

Another example was the removal of promethazine from the formulary and all areas of the hospital due to the risk of tissue injuries (best practice #13). Participants did not know the risks of using it or the reasons behind removing it from the hospital.

“Yes, promethazine is found in the hospital. Why are we supposed to remove it?” P4, Interview (IV) 1.

Motivation

Lack of motivation to implement the best practices was one of the challenges for non-implementation. For example, one participant mentioned that regulatory affairs not requiring the implementation of the ISMP best practices (e.g., best practice #13) could be one of the reasons for non-implementation.

“We only follow the ISMP in the things that are asked by us from the national board for accreditation of healthcare institutions such as tall man lettering, look-alike and sound alike, and fall risk medications.” P4, IV1.

Another factor that was mentioned is the personal interest in a specific topic. For example, one participant mentioned that, although it was not required by the hospital, their personal interest in medication safety encouraged them to seek information from other organizations to prevent errors from happening in their organization (best practice #14).

“I think this is something more personal, for me I am in several groups that discuss this topic, but others may not have the same interest.” P3, FG1.

Opportunity (Lack of Resources)

Lack of resources such as protocols, materials, or space was one of the commonly mentioned challenges that participants believed hindered their ability to implement best practices. Not having a specific protocol or a team in charge of pain management tasks were some of the mentioned challenges for not verifying or documenting opioid stats and type of pain for patients (best practice #15).

“Not having a committee related to pain management makes it difficult for us to implement these requirements.” P5, IV2.

“There are no protocols for situations like these, so it is not implemented.” P1, FG1.

The lack of space in the pharmacy was another reason for non-implementation. When asked for reasons behind not separating neuromuscular blocking agents from other medications (best practice #7), participants believed it was due to a lack of space.

“Yes, you are correct [participant replying to the researcher asking whether it’s a space issue] because we don’t have enough space], do you agree, doctor [participant P2 asked another participant P3]? Yes, I agree.” P2 and P3, FG1.

Another resource issue was the lack of required materials to implement the best practices. For example, participants were aware that dispensing vinca alkaloids in a mini bag rather than a syringe is a best practice (best practice #1). However, they did not implement it due to the lack of mini bags.

“We know this is a best practice but it’s not applicable in this hospital because we don’t have the mini bags in the hospital.” P2, FG1.

The same issue was mentioned with dispensing oral liquid medications in an oral syringe rather than a parenteral syringe that can mistakenly connect to a vascular access line (best practice #4).

“Not applicable because we don’t have the syringes mentioned, we need to add them to the formulary.” P2, FG1.

One of the important opportunities that affected implementation was the electronic system used in the hospital. A system that does not support the implementation of the best practices or does not have some features was highlighted by many participants. For example, when discussing challenges related to using a default weekly regimen for oral methotrexate in the electronic system (best practice #2), participants believed it was because of the current electronic system.

“It’s not implemented you see; this is my field because I’m the one who does the setup. Mainly because we don’t have the option to have a default order in the system. The only time its default is when it’s a protocol such as chemotherapy protocol.” P4, IV1.

“As a physician, there are no default orders in the system for methotrexate, the physician must write down the required dose themselves.” P1, FG1.

## Discussion

Our study analyzed the implementation status and challenges associated with non-implementation of the ISMP best practices in a tertiary hospital in SA. Highest implemented best practices were best practices #9, #11, #16, and #19. While least implemented best practices were best practices #4, #5, #6, #10, and #18. Challenges to implementation were related to knowledge, motivation, and opportunity, where the latter was found to be the highest theme associated with non-implementation.

Four best practices were implemented the most in the hospital. When comparing the highest implemented best practices in our institution to the 2021 ISMP survey, we found that having antidotes and reversal agents readily available (best practice #9, 75%) and limiting medications that can be removed from the automated dispensing unit by override (best practice #16, 75%) had similar implementation rates in the ISMP survey (74% and 75%, respectively) [12]. For the independent verification of sterile preparation (best practice #11), we had a high implementation rate (75%). Many sterile preparations are high-alert medications, which are “drugs that bear a heightened risk of causing significant patient harm when they are used in error” [16]. These medications bear many risks such as lack of standardized concentration, lack of use of current technology solutions, and poor environment [17]. The findings from our study are similar to published evidence in SA. One study across hospitals in SA found that written policies for sterile preparations were available and implemented [18]. However, when compared to the ISMP survey, they had a low implementation rate (34%) [12]. Their explanation was that their focus was on implementing this best practice on high-alert medications and not for all sterile preparations. As for the newest best practice related to having strategies to improve safety with high-alert medications (best practice #19), no data was available from the ISMP regarding the implementation status, as it is a new best practice that was added in 2022-2023. However, in a baseline survey in 2022, the ISMP found that there is still room for improvement regarding the implementation of the three new best practices (#17-19) [13].

Three themes were identified related to challenges for implementation, namely, knowledge, motivation, and opportunity. Knowledge can be classified into knowing about the existence of the best practices, knowing about the rationale behind the best practices, knowing about the importance of these best practices in terms of reducing errors and harm to patients, and knowing about the implementation process of the best practices. People might know that something is better done in a certain way. However, they might not know why. Evidence shows that knowledge is one of the important factors that affect the performance of healthcare providers and the quality of care they provide [18-20]. Findings from the ISMP survey did not include knowledge as one of the challenges/barriers to implementation [12]. However, in our study, knowledge was one of the barriers that affected the implementation of some best practices such as best practices #10 and #13. Participants were not aware that some of the practices were better done in the way suggested by the ISMP. For example, the elimination of all 1,000 mL bags of sterile water from all areas of the hospital except the pharmacy (best practice #10) had a low implementation rate (37.5%). The practice was recommended to reduce the accidental administration of an intravenous infusion of sterile water, which may lead to the destruction of red blood cells (hemolysis) and cause severe harm to patients. Participants were not aware of the rationale behind removing it from other areas of the hospital, wondering why sterile water should be removed. One explanation is that managers responsible for certain areas might focus on best practices related to their area, rather than general best practices in hospitals. Another explanation is that they might believe that safety is the responsibility of the medication safety officer. No one was holding this position at the time of the study. This might explain the lack of knowledge of other staff of safety-related resources such as the ISMP.

The best practice related to eliminating injectable promethazine from the hospital (best practice #13) had a very low implementation (12.5%). Promethazine is a medication that is commonly used to treat nausea and vomiting. It is a high-alert medication marked with a boxed warning because it may result in severe tissue damage and adverse reactions if mistakenly given subcutaneously or into the artery. It was on the ISMP list of high-alert medication for the past 16 years because of the high risk of error and adverse reactions and was advised to be removed from the formulary in 2018-2019 [11]. This best practice was a surprise to almost all participants. None of them knew why it was a best practice. One participant asked why we should remove it. Compared to published data, the implementation rate of this best practice was similar to evidence from other hospitals. Data from numerous US hospitals showed that compliance with this best practice was low [21]. In addition, it was one of the lowest-implemented best practices in the 2021 ISMP survey (37%) [12].

Motivation has many meanings. It can mean the feeling of wanting to do something (e.g., personal interest), the need to do something (e.g., when it is required by the hospital), or it could mean the person’s belief that it is good to do something. In our study, lack of motivation was one of the challenges to implementation. As mentioned earlier, eliminating injectable promethazine from the hospital (best practice #13) had a very low implementation (12.5%). Participants followed best practices when they were also required for accreditation and the ISMP best practices are not mandated by any accreditation body in the hospital. This is consistent with published evidence that showed that lack of staff motivation was a barrier to intervention implementation [22-24]. Another motivational issue was personal interest which caused some participants to take the initiative in following some best practices, despite not being explicitly stated as best practice in their hospital policy.

Even if healthcare providers have the knowledge and are motivated to implement these best practices, opportunities in the system (i.e., lack of resources) may hinder their ability to do so. According to the participants, many opportunities were lacking such as a lack of specific protocols, materials such as mini-bags and oral syringes, space in the pharmacy, and unsupportive electronic systems to implement the best practice. For example, the lack of resources contributed to the low implementation rate of two best practices: the use of oral syringes (best practice #4, 12%) and the use of oral liquid metrics (best practice #5, 25%). Prioritizing resources based on the prevalence of error in the hospital could help in implementing best practices.

Lack of system support was another issue that did not allow participants to use a default weekly regimen of methotrexate (best practice #2, 37.5%). Having a flexible system might be useful. However, it is understandable that some hospitals might not have a sophisticated electronic system or an electronic system at all. If a hospital has a pharmacy informatician, then they can consider flexible options when choosing their electronic system. Raising awareness about some of the best practices might also help with the limited resources. For example, dispensing vinca alkaloids in a mini bag rather than a syringe is a best practice to avoid administration via any route other than intravenously. Intrathecal administration of vinca alkaloids is fatal. This information is highlighted in the hematology guidelines and protocol. It is stated that “only Methotrexate, Cytosine (aka Cytarabine) and Hydrocortisone are given intrathecally. Cytosine should never be given on the same day as IV Vincristine” [25]. Healthcare providers might have these guidelines in the hospital. However, it is important that these guidelines and protocols are readily available and accessible to ensure proper administration of vinca alkaloid medication regardless of the availability of mini bags.

For the Targeted Medication Best Practices to be implemented, the three identified themes must be addressed. First, healthcare providers in the hospital must have the capability to do/perform the best practices. For example, they must have the required knowledge, training, skills, etc. to perform the best practice. Second, healthcare providers must be motivated enough to follow and implement the tasks in the way described in the ISMP best practices. Finally, healthcare providers must have the opportunity to implement the best practices in terms of the availability of resources, personnel, time, space, location, and so on.

Safety of medication use is the responsibility of all healthcare providers in the institution, especially those in the pharmacy. However, many healthcare providers believe that the work would be more optimized if it stems from one individual rather than many people assigned different tasks related to medication safety issues [26]. Having a medication safety officer to overlook the safety issues would save time and effort and would facilitate the use of information inside and outside the organization, drive safety improvement, and increase the implementation rate of best practices related to the safe use of medications. Evidence shows that a higher level of implementation was seen in hospitals that have a medication safety officer [26].

Errors occur and will continue to occur because of the complexity of the healthcare system, patients, and medications. Identifying common safety issues in the institution and focusing on best practices that can improve the safety one at a time would help. Future alignment between the hospital committees such as the Pharmacy and Therapeutic Committee and Adverse Drug Reaction Committee with the medication safety officer would be useful in analyzing errors and prioritizing best practices that need to be targeted. In addition, joining forces between hospitals to learn from their experience in implementing the best practices (opportunities, barriers, and misperceptions) would also help in implementation.

Future research could focus on action plans that can be taken by hospitals to implement these best practices. While taking the perspectives of pharmacists, physicians, and nurses into consideration as they might be involved with the implementation. In addition, further work could focus on the most appropriate interventions that can be taken to improve the knowledge of healthcare providers, motivate them to implement best practices, and best ways to improve opportunities in the environment.

Our study has a few strengths. First, to our knowledge, this is the only study in SA that looked at the ISMP best practices and challenges associated with their implementation. Findings from our study may serve as a guide for best practices implementation process in other healthcare organizations. Second, we used mixed methods in evaluating the implementation status of the best practices. We asked open-ended questions in the interviews/focus groups rather than relying solely on the questions of the survey (questions with pre-defined answers). Our method allowed us to explore ideas and challenges and to have a better understanding of the implementation status. Third, we interviewed all healthcare providers holding a managerial position in the pharmacy. We believed that compared to all personnel in the pharmacy, managers would be more aware of the implementation of best practices and challenges that might affect implementation.

This study has a few limitations. First, it was done in a single center, which may affect the generalizability of findings. Second, the sample size was small (eight managers), and we only interviewed five participants. However, all eight participants completed the survey.

## Conclusions

Out of the 19 best practices, only four were fully implemented and one of them was in progress. The low implementation rate was due to barriers related to knowledge, motivation, and opportunity, with the latter being the most common. Hospitals and healthcare providers need to be aware of the best practices and the rationale behind them, motivated to, and have the necessary resources to implement them in their hospitals.

## Appendices

Appendix A: Definition of each best practice

**Table 3:** Definition of each best Practice (adapted from the Institute for Safe Medication Practices). 1: “Three New Best Practices in the 2022-2023 Targeted Medication Safety Best Practices for Hospitals | Institute for Safe Medication Practices.” (2022). Accessed: August 14, 2023: https://www.ismp.org/resources/three-new-best-practices-2022-2023-targeted-medication-safety-best-practices-hospitals. 2: “Survey Shows Room for Improvement with Three New Best Practices for Hospitals | Institute for Safe Medication Practices.” (2022). Accessed: August 14, 2023: https://www.ismp.org/resources/survey-shows-room-improvement-three-new-best-practices-hospitals#:~:text=The%20three%2....

Number of best practices	Description of best practice
1	Dispense vincristine and other vinca alkaloids in a minibag of a compatible solution and not in a syringe
2	Use a weekly dosage regimen default for oral methotrexate in electronic systems when medication orders are entered
	Require a hard stop verification of an appropriate oncologic indication for all daily oral methotrexate orders
	Provide specific patient and/or family education for all oral methotrexate discharge orders
3	Weigh each patient as soon as possible on admission and during each appropriate outpatient or emergency department encounter
	Measure and document patient weights in metric units only
4	Ensure that all oral liquid medications that are not commercially available in unit dose packaging are dispensed by the pharmacy in an oral syringe or an enteral syringe
5	Purchase oral liquid dosing devices (oral syringes/cups/droppers) that only display the metric scale
6	Eliminate glacial acetic acid from all areas of the hospital
7	Segregate, sequester, and differentiate all neuromuscular blocking agents (NMBs) from other medications, wherever they are stored in the organization
8	Administer medication infusions via a programmable infusion pump utilizing dose error-reduction systems
	Maintain a 95% or greater compliance rate for the use of dose error-reduction systems
9	Ensure all appropriate antidotes, reversal agents, and rescue agents are readily available
	Have standardized protocols and/or coupled order sets in place that permit the emergency administration of all appropriate antidotes, reversal agents, and rescue agents used in the facility
10	Eliminate all 1,000 mL bags of sterile water (labeled for “injection,” “irrigation,” or “inhalation”) from all areas outside of the pharmacy
11	When compounding sterile preparations, perform an independent verification to ensure that the proper ingredients (medications and diluents) are added, including confirmation of the proper amount (volume) of each ingredient prior to its addition to the final container
12 (merged with BP 15)	Eliminate prescribing of fentanyl patches for opioid-naive patients and for patients with acute pain
13	Eliminate injectable promethazine from the formulary
14	Seek out and use information about medication safety risks and errors that have occurred in other organizations outside of your facility and take action to prevent similar errors
15	Verify and document a patient’s opioid status and type of pain before prescribing and dispensing extended-release and long-acting opioids
	Default order entry systems to the lowest starting dose and frequency when initiating orders for extended-release and long-acting opioids
	Eliminate the prescribing of fentaNYL patches for opioid-naïve patients and/or patients with acute pain
	Eliminate the storage of fentaNYL patches in automated dispensing cabinets or as unit stock in clinical locations where acute pain is primarily treated
16	Limit the variety of medications that can be removed from an automated dispensing cabinet (ADC) using the override function
	Require a medication order prior to removing any medication from an ADC, including those removed using the override function
	Monitor ADC overrides to verify appropriateness, transcription of orders, and documentation of administration
	Periodically review for appropriateness the list of medications available using the override function
17	Safeguard against errors with oxytocin use
18	Maximize the use of barcode verification prior to medication and vaccine administration by expanding use beyond inpatient care areas
19	Layer numerous strategies throughout the medication use process to improve safety with high-alert medications

Appendix B: Participant information sheet

You are being invited to take part in a research study. Before deciding if you want to take part, we need you to understand the goal of the research and what it will involve. Please read the following information carefully. If you have any questions, please do not hesitate to ask. Thank you for reading this.

What is the aim of the study?

The aim of this study is to assess the implementation status of the ISMP best practices in a tertiary hospital in SA and the barriers associated with non-implementation.

Why have I been invited?

You have been chosen to take part as you are a healthcare provider working in the pharmacy and hold a managerial position in the pharmacy.

What will I have to do?

After taking your consent, we will arrange a short interview or focus group with you (approximately 20-30 minutes). We will present to you the results of the survey and ask a few questions about some of the best practices.

What is the duration of the study?

We expect that data collection will last for two months.

What will happen with the data collected?

Data collected will be analyzed by the researchers. No information that can identify you will ever appear in any report. At the end of the study and per request, we will be happy to offer you a short summary highlighting the main results of the study.

What are the potential benefits and possible risks?

The study will help raise awareness about the targeted medication safety best practices. There are no risks to you in taking part in this study. If you feel the need to further discuss any issues related to the study, please contact any of the researchers using the details provided below.

How is confidentiality maintained?

We assure you of the confidentiality of the data collected. No one outside the research team will know what you have said or that you are participating in the study. The data will only be used for the purpose of study, and the data will be analyzed anonymously.

All information will remain confidential and will not be given to anyone else.

What happens if I do not want to take part or if I change my mind?

It is your choice to decide whether you want to be part of the study. You can withdraw at any time without providing a reason or detriment to yourself. If you withdraw within the data collecting process, all your data will be destroyed. If you decide to withdraw after the data has been anonymized, it might not be possible to identify the information that belongs to you and discard it.

Ethical approval?

This study has received ethical approval from the King Abdul Aziz University Hospital Research Committee.

What if something goes wrong?

If you have a concern about any aspect of this study, you should contact the researcher who will do their best to answer your questions.

Appendix C: Consent form

If you are willing to participate, please complete and sign below.

 I confirm that I have been informed about the information sheet on the above project and have had the opportunity to consider the information and ask questions and was answered satisfactorily.

 I understand that the discussion will be audio-recorded.

 I agree to the use of anonymous quotes.

 I agree to take part in the above project.

__________________________   _________________    _________________________

Name of participant                                        Date                                        Signature 

__________________________   _________________    _________________________

Name of person taking consent                    Date                                        Signature

Appendix D: Interview/Focus group schedule (topic guide)

(Start recording)

You are invited today to take part in a research study to evaluate the implementation status of the ISMP best practices and the barriers associated with non-implementation. This discussion will take approximately 20-30 minutes and will be audio-recorded. All information discussed during this interview will be confidential and only be shared with the researchers of the study. Data will be analyzed anonymously and any identifying information including names of anyone mentioned during the interview will be removed.

As you all know this research is about evaluating the implementation status of the ISMP best practices and what are the challenges you face with implementation. We appreciate you all for taking the time to fill out the survey beforehand. For this discussion, we will go through each best practice while showing you the implementation status for each best practice. What we want from you is to further validate the answers to the survey and explain to us what challenges you are facing in the case of non-implementation.

Thank you for your time and cooperation.

Overview of each discussion

1-    We presented the results for each BP (frequency %) one by one while explaining to them what each best practice is.

2-    Participants were asked to elaborate more on the findings and the presented frequencies.

3-    We asked participants to elaborate more on the challenges associated with implementation.

Thank you for your participation and time.

(Stop recording)

Appendix E: Full survey findings

**Table 4:** Full findings of the survey.

Best practice	Fully implemented	In progress	Not implemented	Not applicable
1	50%	12.5%%	12.5%	25%
2	37.5%	12.5%	50%	-
2b	62.5%	-	25%	12.5%
2c	37.5%	25%	37.5%	-
3	62.5%	-	25%	12.5%
3b	87.5%	12.5%	-	-
7	62.5%	12.5%	25%	-
8	37.5%	50%	12.5%	-
8b	50%	25%	-	25%
8c	37.5%	25%	-	37.5%
8d	37.5%	37.5%	-	25%
9	75%	12.5%	-	12.5%
11	75%	25%	-	-
13	-	12.5%	62.5%	25%
14	37.5%	25%	25%	12.5%
15	62.5%	12.5%	-	25%
16	75%	-	12.5%	12.5%
16B	50%	12.5%	12.5%	25%
16C	62.5%	25%	-	12.5%
16D	75%	12.5%	12.5%	-
17	62.5%	-	12.5%	25%
17-B	50%	12.5%	12.5%	25%
17-C	50 %	-	12.5%	37.5%
17-D	62.5%	12.5%	-	25%
17-E	37.5%	-	37.5%	25%
17-F	62.5%	-	12.5%	25%
18	12.5%	12.5%	50%	25%
18B	62.5%	-	12.5%	25%
18C	25%	25%	25%	25%
19	87.5%	12.5%	-	-
19B	87.5%	12.5%	-	-
19C	62.5%	25%	-	12.5%
19D	62.5%	25%	-	12.5%
19E	87.5%	12.5%	-	-
19F	75%	12.5%	-	12.5%
19G	62.5%	25%	-	12.5%
4 (archived)	12.5%	25%	50%	12.5%
5 (archived)	25%	12.5%	25%	37.5%
6 (archived)	37.5%	12.5%	-	50%
10 (archived)	37.5%	-	37.5%	25%
